# Accelerating Evidence-Informed Vaccine Introductions: Lessons from the Hexavalent Early Adopters Workshop

**DOI:** 10.3390/vaccines14050452

**Published:** 2026-05-19

**Authors:** Kathryn L. Hopkins, Sidy Ndiaye, Zeinebou Sidi Abdullah, Rita Atugonza, Ousseynou Badiane, Khassoum Ba, Tyler Best, Jean Claude Bizimana, Dah Cheikh, Jean Claude Andrianirinarison, Eraste Rwagitare, Tene-Alima Essoh, Nhamo Gonah, Stephen C. Hadler, Benjamin M. Kagina, Leopold Lambou, Abdoulaye Mangane, Wilberforce Musoga Kabweru, Osée Rurambya Sebatunzi, Mohamedhen Itawel Oumrou, Priscylla Volazandry, Lalao Harisoa Ramanandraibe, Noeline Ravelomanana, Theresa Sommers, Lisandro Torre, Elisabeth Wilhelm, Atakouma D. Yawo, Allarangar Yokouide, Ronald Wasswa, Lassane Kabore

**Affiliations:** 1 Sabin Vaccine Institute, Washington, DC 20006, USA; kate.hopkins@sabin.org (K.L.H.);; 2 World Health Organization Regional Office for Africa (WHO AFRO), Brazzaville, Congo; 3 GCEI Secretariat/National Immunization Technical Advisory Group (NITAG), Nouakchott, Mauritania; 4 Vaccines and Immunization Division, Ministry of Health, Kampala P.O Box 7272, Uganda; 5 Immunization Division, Ministry of Health and Public Hygiene, Dakar 10000, Senegal; 6 Regional Health Directorate, Trarza, Mauritania; 7 Expanded Program on Immunization (EPI), Bujumbura, Burundi; 8 School of Medicine, University of Antananarivo, Antananarivo 101, Madagascar; 9 Expanded Program on Immunization (EPI), Kigali P.O. Box 84, Rwanda; 10 African Preventive Medicine Agency, Abidjan, Côte d’Ivoire; 11 National Immunization Technical Advisory Group (NITAG), Harare, Zimbabwe; 12 Task Force for Global Health, Decatur, GA 30030, USA; shadler@taskforce.org (S.C.H.); ltorre@taskforce.org (L.T.); 13 Vaccines for Africa Initiative, University of Cape Town, Cape Town 8000, South Africa; 14 Effective Basic Services Africa (eBASE), Bamenda, Cameroon; 15 Expanded Program on Immunization (EPI), Dakar, Senegal; abdoulayemangane@yahoo.fr; 16 Mulago National Referral Hospital, Kampala P.O Box 7051, Uganda; drwilberforcemkabweru@gmail.com; 17 School of Medicine and Pharmacy, University of Rwanda, Kigali P.O Box 4285, Rwanda; sebatunzi12@gmail.com; 18 Regional Health Directorate, Gorgol, Mauritania; 19 Ministry of Public Health, Antananarivo 101, Madagascar; 20 National Immunization Technical Advisory Group (NITAG), Ministry of Public Health, Antananarivo 101, Madagascar; 21 National Immunization Technical Advisory Group (NITAG), Lomé, Togo; 22 National Immunization Technical Advisory Group (NITAG), N’Djamena, Chad; 23 School of Public Health, Makerere University, Kampala P.O Box 7272, Uganda; 24 Gavi (Former), 1218 Geneva, Switzerland

**Keywords:** hexavalent vaccine, vaccine introduction, immunization systems, Africa, policy analysis, Gavi, implementation, peer learning, health system strengthening

## Abstract

**Background/Objectives**: Transitions to new vaccines or antigen schedules represent complex system changes requiring coordinated governance, reliable data systems, domestic financing, and multisectoral collaboration. In 2025, African countries were moving toward a switch from separate pentavalent and inactivated poliovirus vaccines to the combined hexavalent vaccine. This project report describes the Hexavalent Vaccine Switch Early Adopters Workshop in Dakar, Senegal, which included ten African countries, and its implications for future vaccine introductions. **Methods**: We conducted a practice analysis drawing on structured documentation of plenary presentations, country case studies, interactive problem-solving sessions, and national roadmap exercises. A thematic framework aligned to ten process points for the hexa switch guided synthesis. **Results**: Countries reported shared system vulnerabilities, including coexistence risks of legacy and new vaccine stocks, inconsistent data completeness, under-resourced vaccine safety surveillance, and financing uncertainties. Early adopter countries demonstrated operational feasibility, logistical efficiencies, and opportunities for reducing injection burden. Outputs included a Health System Adaptation Checklist, a Switch Risk Mitigation Catalog, and 12-month national roadmaps. **Conclusions**: Regional peer-learning mechanisms can accelerate decision-making, improve operational quality, and strengthen accountability for vaccine introductions. Structured cross-country collaborations can transform a product switch into a scalable system-strengthening opportunity.

## 1. Introduction

Introducing a new vaccine or modifying an existing immunization schedule is among the most complex transformations national immunization programs undertake [1]. Such transitions require not only technical readiness but also coordinated action across policy deliberations, financing, supply chains, data systems, health workforce capacity, stakeholder buy-in, and sociopolitical advocacy. In the African region, recent progress toward adopting more integrated delivery platforms, including the hexavalent (hexa) vaccine combining diphtheria–tetanus–pertussis, hepatitis B, *Haemophilus influenzae* type b, and inactivated poliovirus vaccine (DTP-HepB-Hib-IPV), has been promising but uneven [2].

Gavi vaccine investments in low- and middle-income countries have expanded protection against multiple vaccine-preventable diseases [3]; however, antigen switches and schedule transitions remain particularly challenging programmatic changes. They require coordinated decision-making, sustainable financing, reliable procurement and distribution systems, updated monitoring tools, and effective communication with health workers and communities. Costing is central to this transition because countries must account not only for vaccine price and co-financing obligations, but also for the operational costs of readiness, including cold chain adaptation, supply chain reconfiguration, health worker training, communication, supervision, monitoring tools, and transition-period stock management. These costs are often distributed across budget lines and partners, making early, comprehensive costing essential for realistic planning, domestic resource mobilization, and sustainable implementation. However, the hexavalent vaccine offers important advantages, such as reduced injection burden, simplified logistics, improved integration of polio vaccination into routine immunization [4], and potential gains in coverage and equity, particularly for underserved populations. Still, countries continue to face persistent gaps in real-world evidence on costs, delivery models, operational readiness, and system effects, and opportunities for structured cross-country learning remain limited.

In 2025, the World Health Organization’s Strategic Advisory Group of Experts on Immunization (SAGE) confirmed that a three-dose IPV schedule beginning at ≥6 weeks provides sufficient immunogenicity [5], reinforcing the programmatic rationale for transitioning from pentavalent plus IPV to hexavalent vaccine. In response, many African countries began evaluating the potential benefits of switching from pentavalent to hexa vaccine but faced uneven readiness across ministries and limited real-world implementation data.

**Figure 1:**
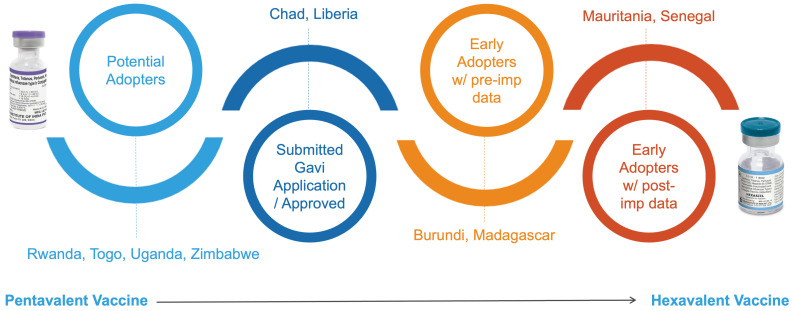
Hexa early adopter workshop participating country switch phases, as of November 2025.

Recognizing these needs, the Sabin Vaccine Institute and the World Health Organization Regional Office for Africa (WHO AFRO) convened the Hexavalent Vaccine Switch Early Adopters Workshop in Dakar, Senegal, an early adoptor of the hexa vaccine, in November 2025. The workshop brought together delegations from ten countries—Burundi, Chad, Liberia, Madagascar, Mauritania, Rwanda, Senegal, Togo, Uganda, and Zimbabwe—representing diverse epidemiological, financing, policy, and health system contexts (Figure 1). Its objective was to strengthen policy and operational readiness for countries at different stages of decision-making, planning, and implementation through direct peer exchange and collaborative problem-solving.

The workshop formed part of a broader shift toward evidence-informed, co-produced learning ecosystems in global immunization programs. The Hexavalent Vaccine Switch Early Adopters Workshop emphasized structured co-creation and multi-stakeholder deliberation to surface cross-cutting system insights. Insights were synthesized from country case studies, peer exchanges, and interactive problem-solving sessions, alongside co-created tools including health system adaptation checklists, risk mitigation frameworks, and country roadmaps. Together, these insights inform improved vaccine antigen switches and introductions in Africa and beyond.

Through this project report, we:

1. Describe the design logic and participatory features of the Hexavalent Vaccine Switch Early Adopters Workshop;
2. Synthesize key insights across decision-making, pre-implementation, and implementation stages of a vaccine switch;
3. Document how regional collaboration mechanisms can promote the quality, efficiency, and sustainability of vaccine introductions;
4. Discuss implications for ministries, partners, and donors, particularly in the context of Gavi 6.0’s shift toward multi-year vaccine budgets and consolidated applications.

## 2. Workshop Design and Methodology

We conducted a practice analysis of the Hexavalent Vaccine Switch Early Adopters Workshop, examining its structure, content, and outputs, designed as a strategic intervention to strengthen immunization decision-making and operational readiness in the African region.

### 2.1. Workshop Design

The workshop gathered approximately 45 participants from ten countries, representing ministries of health, including EPI programs and national immunization technical advisory groups (NITAGs). Technical partners included WHO and UNICEF regional offices, and the Sabin Vaccine Institute-led Hexavalent Vaccine (Switch) Assessment Consortium members (WHO AFRO, Task Force for Global Health, eBASE, Makerere University, AMP Afrique, SOMAPED). The three-day collaborative agenda was thematically structured across the vaccine introduction cycle: decision-making, pre-implementation, and implementation/monitoring. Each session included country-led panels, guided discussions, case studies, group problem solving, and practical tool co-development and use.

### 2.2. Data Sources

Data for this analysis were drawn from:

- **Country case study presentations** from early adopting countries (Mauritania and Senegal, switched on 1 July 2025) and countries in their pre-implementation planning phase, switching in Q1 or Q2 2026 (Madagascar and Burundi), to reflect on and surface diverse approaches, shared challenges, and innovative solutions regarding decision-making, planning, and implementation.
- **Partner technical presentations** on the rationale for switching to hexavalent vaccines, emphasizing disease burden, polio eradication contributions, and updated WHO SAGE recommendations; Gavi co-financial and Gavi 6.0; and World Bank macro-fiscal analysis and strategies for sustainable financing.
- **Ten “process point” discussions** spanning governance and policy, demand forecasting and procurement planning, logistics management, financing, health worker training and supportive supervision, immunization service delivery, communication and social mobilization, program management, monitoring, evaluation and learning/data and reporting, and vaccine safety surveillance.
- **Crowdsourced outputs:**
  - Health System Adaptation Checklist: A practical tool to translate country experience into stepwise time-bound actions that national teams can use to strengthen readiness.
  - Switch Risk Mitigation Catalogue: A consolidated set of country-level vulnerabilities and solutions across process points to provide a foundation for refining preparedness activities.
- **Country roadmaps** developed to transform broad workshop learnings into concrete, time-bound national plans that could be taken forward for internal validation and partner coordination. The session bridged dialog to action, equipping countries with a structured roadmap to guide technical, operational, and financial preparedness for hexavalent vaccine introductions.
- **Collaborative notes and plenary summaries**
- Anonymous pre-, during, and post-**workshop interactive polling** (via Menti) of delegate expectations, questions, workshop experiences and feedback.

All data were generated during the workshop and compiled in bilingual documentation. No personal or sensitive data was collected.

This manuscript’s co-authors represented countries and organizations participating in the workshop and contributed to synthesizing the workshop’s above outputs.

### 2.3. Analytical Framework

We applied a directed thematic analysis aligned to the ten process points defined in the Hexa Switch Assessment Framework, developed for this workshop by adapting WHO’s health system building blocks [6], grouped into three operational phases:

1. Decision-making, financial planning, and governance;
2. Pre-implementation system strengthening;
3. Implementation and monitoring.

Thematic synthesis was conducted to identify patterns of risks and barriers and solutions.

### 2.4. Ethical Considerations

This manuscript reports proceedings and discussions from a technical workshop and does not present findings from human subjects research; therefore, institutional ethics review was not required. All workshop participants contributed in their official professional, non-research capacities, who provided informed consent for workshop documentation, including that discussions and outputs would be synthesized for learning and publication purposes. No identifiable data are reported.

### 2.5. Artificial Intelligence Use Statement

During the preparation of this manuscript, artificial intelligence (AI) tools, including OpenAI ChatGPT (GPT-5.1), were used to assist in compiling workshop notes and data collected during the Hexavalent Vaccine Switch Early Adopters Workshop and to generate structured summaries of workshop inputs. Workshop presentations were also reviewed using AI-assisted tools to cross-check content against workshop notes and support the development of comprehensive summaries and records. French-language sessions and presentations were translated into English using DeepL SE DeepL Translator and subsequently cross-checked by a native French speaker.

Initial thematic synthesis and interpretation were conducted by the workshop organizers. Outputs were subsequently reviewed using AI-assisted tools to support consistency, completeness, and organization. AI was also used for language editing and formatting support. All final analytical judgments, interpretations, and manuscript content were reviewed and approved by the authors, who take full responsibility for the final manuscript.

## 3. Results: Workshop Outcomes and Key Learnings

**Figure 2:**
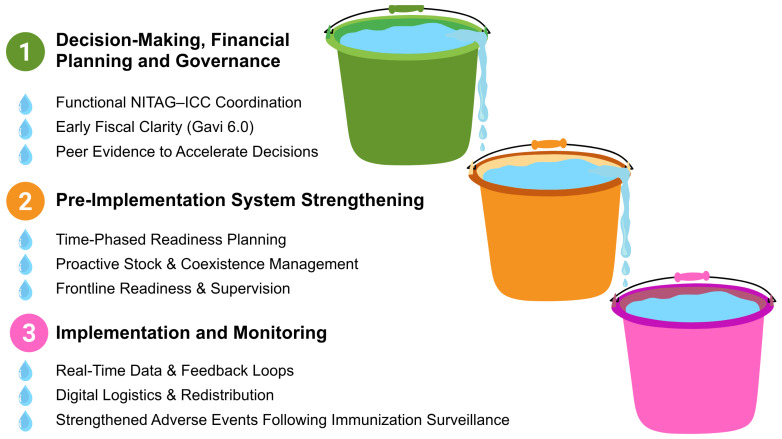
Enabling factors identified across three vaccine planning phases from country discussions for successful vaccine switches.

Although the workshop’s ten participating countries represented diverse contexts, their experiences illuminated similar opportunities and vulnerabilities that determine the success of vaccine switches or introduction within national immunization programs (Figure 2). Their experiences and discussions are summarized by the implementation phase and process point below.

### 3.1. Decision-Making, Financial Planning and Governance

Strong program management was essential for translating hexavalent policy decisions into immunization delivery sessions, although the capacity to manage logistics and stocks adequately varied. The trajectory toward the switch was strongly influenced by the maturity of national governance structures, particularly NITAGs and Inter-agency Coordination Committees for Immunization (ICCs), which serve as foundational mechanisms for evidence appraisal, policy endorsement, and coordination. Senegal’s established, well-functioning bodies, including ICC technical, communication, logistics and supply chain, finance and surveillance sub-committees, smoothed the path from policy to rollout: its NITAG deliberated and endorsed the hexa switch plan presented by the ICC within weeks and specialized committees for logistics, pharmacovigilance, and communication helped mitigate implementation challenges, enabling timely Gavi application submission.

In contrast, countries with less institutionalized governance and program management structures faced delays and coordination gaps. Madagascar required additional time to conduct evidence-to-recommendation reviews, ensure cross-ministerial alignment, and navigate procedural requirements. Mauritania’s experience underscored the importance of inclusive planning, as early gaps in communication between central and peripheral levels hampered initial rollout. Burundi employed a highly structured governance framework, requiring sequential NITAG, ICC, and ministerial approvals, which ensured technical rigor but reduced responsiveness, especially amid compressed timelines. Both Madagascar and Burundi showed that even well-designed governance systems can be strained when multiple new vaccines or child health initiatives coincide, stretching limited management capacity.

Countries reported heavy reliance on partner support to compensate for capacity constraints, underscoring the need for clearer division of roles, more predictable technical assistance, and realistic implementation timelines. All countries experienced financial planning challenges amid an uncertain global funding landscape for health systems and immunization programs. This was compounded by widespread uncertainty about how co-financing obligations would evolve under Gavi 6.0, particularly as countries confront the transition to fixed, multi-year Country Vaccine Budgets. Delegates expressed serious concerns about navigating changing vaccine financing expectations from GAVI in light of updated SAGE recommendations, whilst integrating potential second-year-of-life booster doses into domestic budgeting cycles and about synchronizing financial approvals with programmatic timelines. World Bank and Gavi presentations provided clarity around macro-fiscal constraints and Gavi 6.0 co-financing implications, underlining that countries must increasingly anticipate and manage fiscal constraints alongside operational ones. The Gavi 6.0 strategy, operational from 2026 to 2060, requires countries to increase their financial contributions to vaccine procurement and immunization program operations to improve sustainability and readiness for transition from Gavi support. This early transparency was particularly valuable for application-stage countries (Chad, Liberia, Uganda, Rwanda) seeking clear pathways to endorsement. This session served as a policy clarifier, providing ministries with up-to-date guidance and helping clarify vaccine cost differentials.

### 3.2. Pre-Implementation Readiness and System Strengthening

#### 3.2.1. Demand Forecasting and Procurement Planning

Key challenges were difficulty obtaining accurate population denominators due to outdated census data; tension between planned introduction dates and actual supply availability; and managing the co-existence of penta/IPV and hexa stocks and cold chain at the regional or facility level (Table 1).

Senegal avoided vaccine coexistence entirely by closely monitoring stock through Logistimo, a real-time stock management system, and canceling outstanding penta/IPV orders early, withdrawing all penta before hexa introduction date. Mauritania’s experience, by introducing hexa for infants under 12 months while using remaining penta stocks for older cohorts or catch-up campaigns, demonstrated how large inherited stock volumes can complicate supervision and monitoring during transitions. Countries undergoing catch-up campaigns (e.g., Big Catch-Up [BCU]) faced added complexity, as these efforts influenced introduction timing.

#### 3.2.2. Logistics Management, Including Cold Chain and Infrastructure

The replacement of two vaccines with a single combined product reduced volumetric burden, removing cold chain capacity as a limiting factor for most countries. However, logistics performance varied markedly due to inventory accuracy and last-mile distribution challenges. In Senegal, national-level cold chain utilization remained well within capacity, utilizing the OPTIVAC push model [7]. Despite adequate storage capacity, Mauritania experienced limited transport availability, maintenance deficits, and underutilization of digital logistics reporting tools. Madagascar and Burundi anticipate modest infrastructure needs but emphasize the importance of accurate inventory management and distribution planning. Collectively, logistical success hinged most on system discipline, redistribution capacity and data quality.

#### 3.2.3. Health Worker Training and Supportive Supervision

Training quality emerged as a determinant of operational effectiveness. All countries used cascade training models, yet their quality and effectiveness varied. Senegal’s multisectoral approach included journalists, community influencers, and religious leaders. Mauritania’s early and structured training deployment strengthened readiness but exposed gaps in thorough comprehension at downstream cascade levels. Madagascar’s hybrid approach to training (digital and in-person) is likely to face connectivity challenges, potentially limiting reach. Across settings, supportive supervision beyond initial training was repeatedly identified as essential for preventing administration errors, ensuring proper stock handling, and detecting early signs of caregiver confusion.

#### 3.2.4. Immunization Service Delivery Implementation

Service delivery strategies reflected differing contexts. Senegal executed a clean-cut withdrawal of penta/IPV prior to the hexa introduction launch date, integrating hexa into routine platforms and updating all management tools concurrently, ensuring all children who started on penta would complete their schedules with hexa. Mauritania adopted an age-phased strategy, reserving penta/IPV for older children involved in BCU campaigns, which increased supervision demands and affected reporting consistency. Burundi plans to emphasize strict avoidance of dual administration and align with child health platforms. Madagascar plans for staggered regional introduction, anticipating challenges from regional uneven penta/IPV stock distribution. Countries expressed the need to update programmatic tools and microplans well ahead of rollout, as delays in tool revision can cascade into data gaps and frontline confusion.

#### 3.2.5. Communication and Social Mobilization

Communication was consistently described as a system amplifier, either enabling a smooth transition or introducing risk. Senegal’s strong regional social mobilization task force used proactive, consistent messaging emphasizing reduced injection burden, preventing misinformation and building confidence (campaign motto: “Less injections, more protection!”). Mauritania implemented a large-scale, comprehensive multilingual communication strategy, engaging parliamentarians, religious leaders, and community networks. Madagascar and Burundi, though equipped with strong communication plans, anticipate facing slow communication material validation cycles, low funding availability, and the risk of misinformation spread, especially in hard-to-reach areas with limited media penetration. Participants widely agreed that consistent, proactive communication was critical, requiring that communication systems must be sustained beyond the initial introduction window to maintain confidence.

#### 3.2.6. Data Systems, Monitoring and Evaluation, and Reporting

Data quality and completeness emerged as major cross-cutting vulnerabilities, even amongst countries with strong digital ecosystems, and were exacerbated during periods of health worker strikes or stock transitions. Senegal’s use of DHIS2 (District Health Information Software 2) and Logistimo enabled proactive logistics monitoring and timely corrective action. Mauritania and Madagascar described irregular data review meetings, inconsistencies in stock reporting, and limited capacity for data triangulation and underuse of digital tools. Pre-launch priorities included integrating hexa-related reporting indicators into DHIS2 and paper tools to reduce data discrepancies. Country experiences reveal that antigen switches, supported by routine data quality reviews, can help strengthen monitoring, evaluation and learning (MEL) systems broadly and especially at the subnational level.

#### 3.2.7. AEFI (Adverse Events Following Immunization) Surveillance and Reporting

Widely acknowledged as a systemic weakness requiring renewed investment, countries identified pervasive underreporting of AEFIs. Although Senegal maintained strong accountability structures for serious AEFI reviews, including weekly bulletins, delays and underreporting persisted for minor events. Mauritania had facility-level data gaps and limited availability of reporting tools and limited provider motivation to document minor AEFI. Madagascar and Burundi, both in pre-introduction phases, want to focus on reinforcing investigation protocols and ensuring timely communication pathways between district and central levels and recently, Zimbabwe has transitioned to digital AEFI reporting. The hexa switch thus functioned as an opportunity for revitalizing broader pharmacovigilance systems.

### 3.3. Implementation Insights and Monitoring

Mauritania and Senegal, the first African countries to introduce the whole-cell pertussis-containing (WP) hexa vaccine, co-financed with Gavi and obtained vaccines through the UNICEF procurement system, provided the workshop’s most valuable empirical insights. Both countries introduced the hexavalent vaccine around 1 July 2025 and shared their experience from the first three months of implementation. While separate manuscripts will communicate results from planned post-introduction evaluations (PIEs) in these countries, the following sections briefly summarize the impact.

#### 3.3.1. Real-World Evidence: Mauritania

In Mauritania, 104,703 doses of hexavalent vaccine were administered in the first three months following national launch. Prior to the switch (January–June 2025), routine immunization performance was declining modestly, with coverage for penta1 and penta3 falling by more than 10 percentage points over this period; IPV coverage showed a similar downward trend. Dropout between first and third doses remained moderate (approximately 15–17 percentage points) but increased toward the end of the pre-switch period.

**Figure 3:**
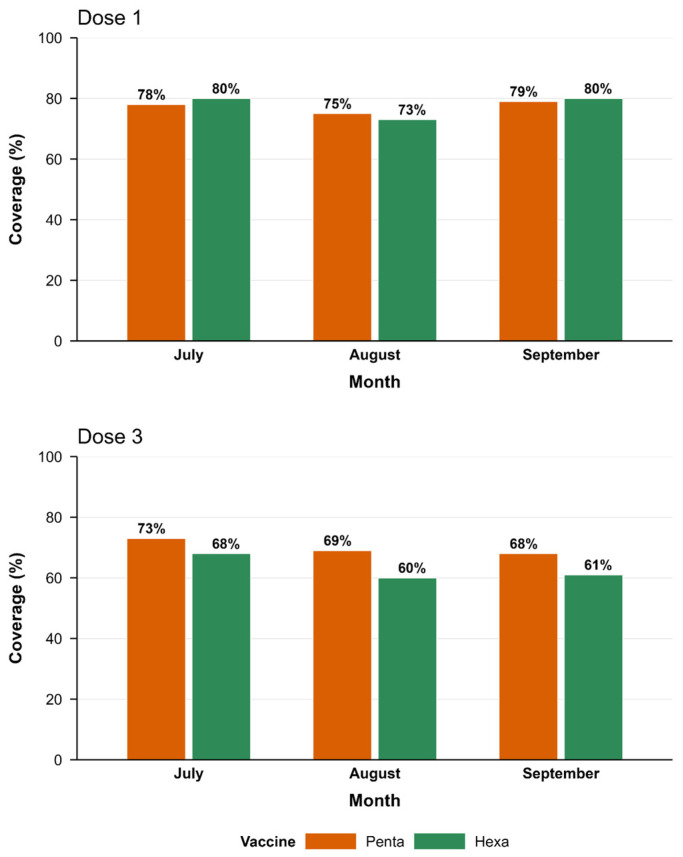
Pre- and Post-Hexa Switch Vaccine Coverage Rates in Mauritania (January–September, 2025).

**Figure 4:**
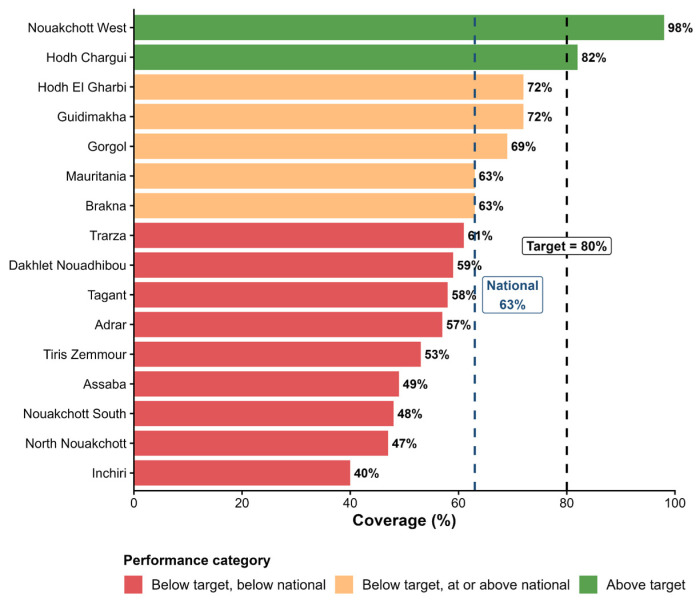
Comparison of cumulative Hexa3 vaccination coverage rates vs. set targets across 15 moughataas in Mauritania.

Following the introduction, early hexa rollout was uneven across regions and districts, with cumulative coverage in the first three months remaining below planned targets. Coverage for the first dose remained relatively stable, fluctuating around 75–80%, indicating that the switch did not disrupt initial service delivery. In contrast, third-dose coverage was lower and more variable (approximately 63–77%), consistent with historical seasonal declines observed during the rainy season, when population mobility and access constraints typically reduce coverage by 1–5 percentage points. Variations in coverage were observed comparing the pre- and post-switch vaccine coverage rate trends (Figure 3) and in comparison of hexa3 vaccine coverage to set targets across all 15 moughataas (Figure 4).

Vaccination coverage varied across urban, rural, and hard-to-reach areas, but patterns were not strictly geographic. Some urban settings underperformed relative to rural areas, while several remote moughataas achieved comparatively strong coverage, reflecting the influence of local factors such as access, staffing, supervision, and community engagement rather than location alone. Health workers reported strengthened monitoring and supportive supervision during rollout, improved staff motivation, and Supply chain performance remained strong, with simplified logistics and uninterrupted vaccine availability. increased uptake of the second dose of measles–rubella vaccine (RR2). Acceptance among parents and caregivers was high, with a clear preference for fewer injections. No vaccine stockouts or unexpected wastage were reported. Key areas for strengthening include improving coverage consistency, reducing dropout (approximately 19%), and reinforcing AEFI surveillance.

#### 3.3.2. Real-World Evidence: Senegal

**Figure 5:**
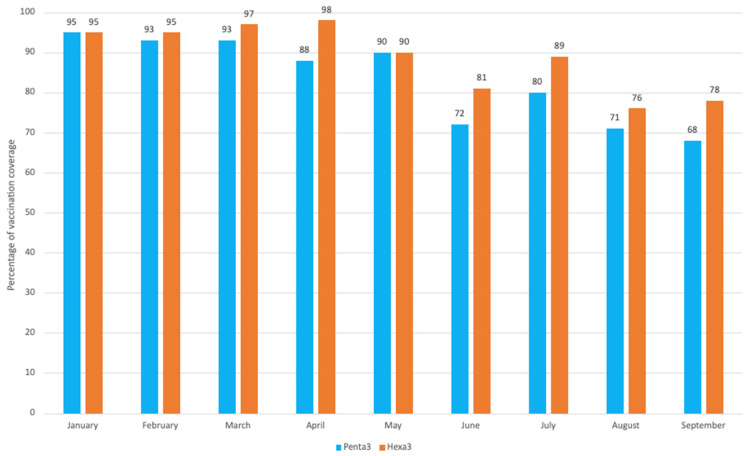
Trends in monthly Penta3/Hexa3 vaccination coverage in Penta3 (2024) and Hexa3 (2025) at the national level in Senegal.

Prior to the switch (January–June 2025), routine immunization performance in Senegal remained strong, with high national penta3 coverage. Following the introduction of the hexavalent vaccine in July 2025, hexa3 coverage reached approximately 89% by September, meeting national targets with reported completeness of 92%. This represented an improvement of roughly 5–10 percentage points compared with penta3 coverage during the same period in 2024. Continuity of vaccination was maintained, with a negative dropout rate between first and third doses (−3%), indicating no attrition associated with the schedule change. During the first three months of rollout, approximately 456,500 hexa3 doses were administered, reflecting rapid national scale-up capacity (Figure 5). Subnational performance was generally consistent across regions. There is no evidence that the switch exacerbated disparities between genders, urban, rural, or hard-to-reach areas. First-dose coverage remained high (hexa1 approximately 85%), suggesting that the switch did not introduce new access barriers.

From a safety and supply chain perspective, implementation was stable. No serious AEFIs were reported during the early post-introduction period, and open-vial wastage remained low (approximately 4%). Although global supply constraints required segmentation of planned deliveries into four shipments, digital logistics platforms enabled real-time stock monitoring and redistribution, preventing stockouts at all levels of the system. The switch generated measurable operational efficiencies. Cold-chain requirements were substantially reduced, with hexa vaccines occupying approximately 41 m^3^ against an available national capacity exceeding 98 m^3^. Simplification of vaccine presentations and injection materials reduced logistical complexity and workload at district and facility levels. Health workers reported time savings, fewer injections per child, and improved caregiver experience.

Overall, Senegal’s experience demonstrates that, in a well-functioning immunization system, the hexavalent vaccine switch can be implemented with minimal disruption, sustained high coverage, and clear efficiency gains, underscoring the importance of system readiness, digital logistics, and coordinated program management in supporting successful antigen transitions.

### 3.4. Co-Created Tools and Outputs

A major workshop contribution was the co-design of a structured set of tools intended to translate regional learning into practical, country-led action. Building on real-world experiences from early adopting countries and systematic analysis across immunization system domains, participants jointly developed three complementary outputs that address recurrent risks and operational bottlenecks associated with the hexa vaccine switch (linked in Data Availability Statement).

First, the workshop generated a Hexa Switch Risk Mitigation Catalog, synthesizing vulnerabilities and solutions identified across country case studies, problem-solving journeys, and thematic discussions. Countries used the catalog as a reference to prioritize risks, inform national preparedness planning, and guide requests for targeted technical assistance.

To complement the risk mitigation catalog, the delegates then co-created a Health System Adaptation Checklist, designed as a comprehensive, time-phased planning tool for vaccine transitions. Drawing on the ten “process points” highlighted throughout the workshop, the checklist organizes concrete actions into three preparatory phases: 9–12 months, 4–8 months, and 1–3 months prior to the switch. This structure reflects the reality that vaccine switches are not discrete events but sequenced system adaptations where checklist items were intentionally framed as observable actions (e.g., “update DHIS2 indicators,” “validate stock withdrawal protocols,”) to support accountability, monitoring, and practical use at all levels.

Third, countries developed a country-specific 12-month roadmap for hexa vaccine introduction, optimization, or post-introduction evaluation, although some countries planned introduction beyond 12 months and focused their roadmap on early-stage planning. These roadmaps articulate clear priorities, milestones, dependencies, responsible actors, and resource requirements across the full introduction cycle. The roadmap embedded workshop insights, including checklist actions and risk mitigation strategies, into existing national planning and budgeting processes, rather than creating parallel plans.

**Figure 6:**
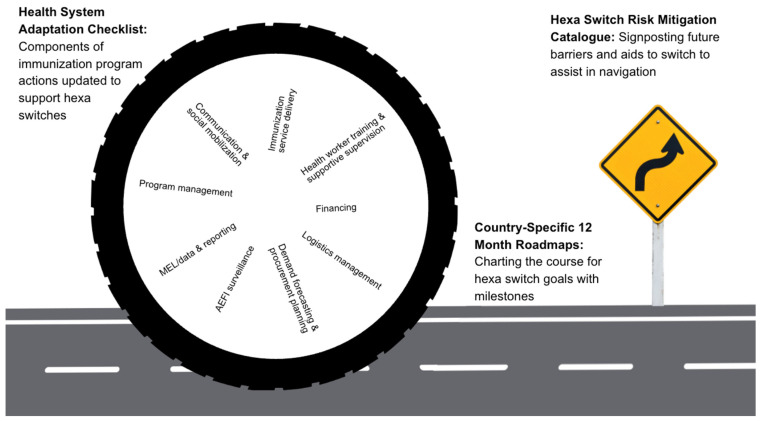
Illustration of co-designed tools supporting hexa switch planning and implementation progress.

Together, these tools form an integrated planning ecosystem collectively functioning as shared and harmonized public goods (Figure 6). The risk mitigation catalog informs proactive management of vulnerabilities; the checklist supports systematic readiness assessment and sequencing of actions; and the national roadmaps translate learning into time-bound, accountable implementation plans.

### 3.5. Key Lessons for Countries Planning a Hexavalent (Hexa) Vaccine Switch

Experience from early adopter countries, including Mauritania and Senegal, highlights a set of practical and transferable lessons for ministries of health and partners preparing to transition from pentavalent plus IPV to the hexavalent vaccine. While implementation contexts differed, successful transitions were consistently underpinned by early planning, strong governance, reliable logistics, effective communication, and rigorous monitoring.

#### 3.5.1. Anticipate Planning and Multisectoral Coordination Early

Countries that established a dedicated technical introduction committee well in advance of rollout were better positioned to manage the complexity of the switch. Effective committees included representation from immunization program management, logistics and cold chain, training, surveillance and AEFI, communication, and key technical and financial partners. Developing a realistic, time-bound national introduction plan supported shared accountability, timely decision-making, and smoother coordination across system levels.

#### 3.5.2. Ensure Predictable Financing and Sustained Political Commitment

Early advocacy with ministries of finance and economy, as well as parliamentary groups on vaccination, was critical to secure co-financing and sustain political buy-in. Clear planning for operational costs and for the second-year-of-life booster dose reduced uncertainty, enabled timely approvals, and minimized delays during implementation.

#### 3.5.3. Secure Vaccine Supply and Inputs While Minimizing Overlap Risks

An accurate inventory of existing pentavalent and IPV stocks prior to introduction was essential to avoid shortages or coexistence at the facility level. Senegal aligned the introduction date with stock depletion timelines and reduced supervision burden, simplified reporting, and avoided confusion during the transition. Proactive stock monitoring and redistribution helped maintain uninterrupted vaccine availability despite global supply constraints.

#### 3.5.4. Invest in Practical Capacity Building and Early Supportive Supervision

Cascade training conducted before introduction, complemented by hands-on sessions on vaccine handling, administration, storage, and communication with caregivers, strengthened frontline readiness. Close and supportive supervision during the first weeks after rollout reinforced correct practices, increased provider confidence, and helped identify and correct early implementation gaps.

#### 3.5.5. Strengthen Communication and Community Engagement Throughout the Transition

Clear, harmonized messages explaining that the hexavalent vaccine replaces pentavalent plus IPV—without adding injections—were central to maintaining caregiver trust and acceptance. Engagement of community leaders, religious authorities, local media, and civil society actors helped prevent rumors, reinforce confidence, and sustain uptake beyond the initial launch period.

#### 3.5.6. Establish Rigorous, Responsive Monitoring and Feedback Loops

Routine use of monitoring tools, including DHIS2, supervision checklists, and electronic stock management systems (e.g., Logistimo, eSMT), enabled rapid identification of operational challenges and areas of low coverage. Systematic review of early implementation data supported timely course correction and continuous improvement.

#### 3.5.7. Coordinate Partner Support Around Nationally Defined Priorities

Targeted and well-coordinated support from technical and financial partners—channeled through national technical committees and introduction working groups—strengthened capacity without fragmenting leadership or duplicating effort. Alignment of partner inputs with country-generated roadmaps enhanced implementation coherence and sustainability.

## 4. Discussion

### 4.1. Workshop Learnings

The Hexavalent Switch Early Adopters Workshop focused on the African continent but it follows the experiences of more than 56 countries that use hexa vaccines globally. Key country findings from hexa switch countries primarily outside of Africa demonstrated that hexa vaccines improved immunization compliance, timeliness and coverage. Global lessons include considering vaccine availability, vaccination schedules, health system readiness, HCP training, vaccine acceptance and infrastructure and resources in advance of hexa switches [8]—themes that also emerged in the Early Adopters workshop.

In Senegal, the Hexavalent Vaccine Switch Early Adopters Workshop functioned as an effective regional system-strengthening mechanism, enabling countries to clarify policy pathways, co-create implementation tools, and collectively address shared operational risks. Although participating countries are at different stages of the hexa introduction journey, their experiences converged around a set of core themes that hold broad relevance for vaccine introductions across Africa and similar contexts.

First, the workshop underscored that antigen switches act as system-wide stress tests, revealing both strengths and latent vulnerabilities within national immunization programs. Delays reflected deeper challenges related to cross-ministerial coordination, evidence appraisal, and financial governance. Readiness gaps in forecasting, logistics, data systems, monitoring and evaluation, and AEFI surveillance were not unique to the hexa switch but symptomatic of broader system constraints. For example, challenges many EPI programs face with “last mile logistics” also can affect hexa switch, risking efforts to leverage hexa to reduce the number of zero-dose children. Through structured, comparative peer exchange, the workshop enabled countries to self-diagnose challenges and identify feasible, context-appropriate mitigation strategies. One participant shared that the workshop “was very informative in terms of sharing experiences between different countries at different levels of preparedness.”

Second, the findings highlight peer learning as a catalyst for accelerated and higher-quality decision cycles and implementation. Countries benefited from real-time access to peers navigating similar challenges. Understanding how another country resolved a specific operational issue, such as Senegal’s management of stock coexistence or Mauritania’s multilingual communication strategy, was more actionable than generic technical guidance. This approach can shorten learning curves, accelerate Gavi application preparation, and inform planning. This experience aligns with evidence from other global learning collaboratives and structured learning networks [9,10], echoing WHO’s evidence-to-policy-to-practice translation approaches, which emphasize country deliberation, iterative feedback, and co-identification of solutions. Delegates commented that they found the interactive model “very informative” allowing “a real exchange of experiences” and several delegations requested that organizers “continue this model.”

Third, the workshop demonstrated how south–south collaboration can accelerate equity-oriented implementation. Early adopters shared practical strategies for reaching hard-to-reach populations, managing seasonal mobility, adopting new digital solutions, and sustaining caregiver trust during transitions. By reducing injection burden, simplifying logistics, and strengthening integrated delivery platforms, the hexa switch has the potential to improve uptake among populations historically underserved by routine immunization systems. However, these equity gains are not automatic but they depend on sustained investments in last-mile delivery, robust supervision, and responsive data systems. Mauritania’s experience illustrates how system volatility and denominator uncertainty can undermine coverage, while Senegal’s strong baseline systems can translate innovation into measurable performance gains. These contrasting cases reinforce the value of regional learning spaces that allow countries to learn from one another. One country’s delegation shared that, “We intend to introduce the hexavalent vaccine after better preparation based on the experiences of our colleagues who have already introduced it.”

Fourth, the analysis points to harmonized partner engagement as increasingly crucial under Gavi 6.0. The transition to fixed Country Vaccine Budgets, consolidated applications, and streamlined digital platforms requires ministries of health to exercise greater foresight, internal coordination, and financial discipline, avoiding duplication. Discussions highlighted the risk of fragmented technical assistance and parallel processes if partner support is not aligned with national priorities. Co-designed outputs offer concrete mechanisms to operationalize alignment, ensuring that partner inputs reinforce rather than dilute country ownership. Importantly, participants emphasized the need for longer-term investments in MEL systems, rather than one-off supports.

Several common data gaps emerged as persistent constraints on evidence-informed decision-making, including limited availability of real-world cost and cost-efficiency data to inform national advocacy and budget negotiations, insufficient evidence on social acceptance and caregiver perceptions beyond initial rollout, and gaps in documentation of delivery models that work best in diverse geographic and demographic contexts. These gaps complicate national advocacy with ministries of finance, constrain prioritization within fixed vaccine budgets, and limit the ability to adapt delivery strategies. Addressing these evidence gaps can sustain hexa implementation and inform future vaccine introductions. Key country priorities should be institutionalizing monthly data review meetings, enhancing inventory accuracy and last-mile logistics, prioritizing AEFI system strengthening as part of routine EPI and leveraging PIEs following implementation to course-correct rollout.

**Figure 7:**
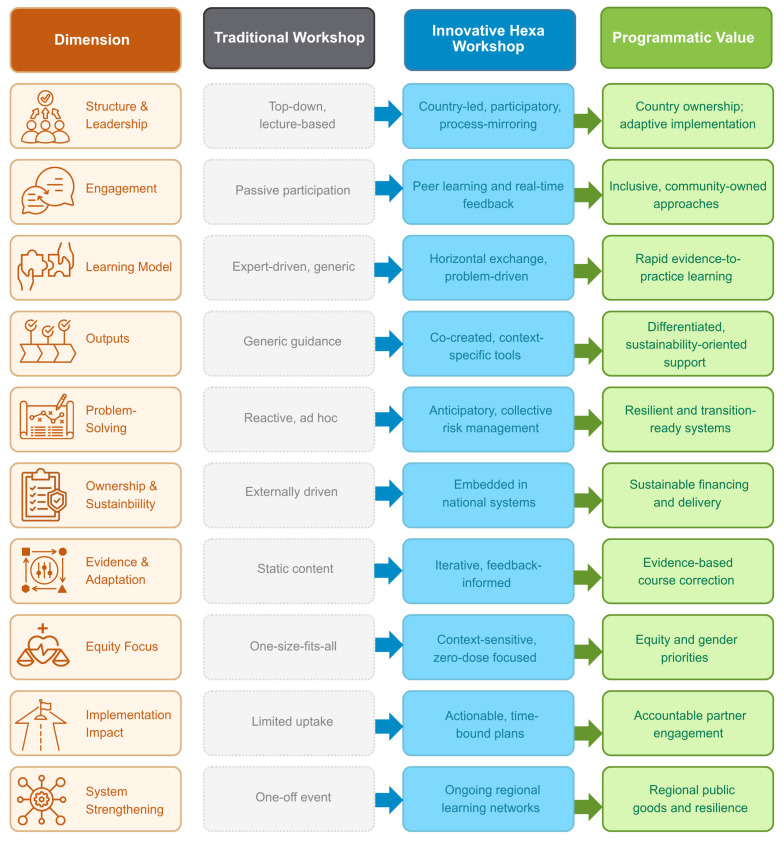
Hexavalent vaccine switch early adopters workshop approach and programmatic value, especially relevant for GAVI 6.0.

Finally, the workshop model of a combination of in-person peer exchange, structured problem-solving, and co-creation of practical, context-sensitive tools provides a blueprint for future introductions and/or updated vaccination schedules of other vaccines, as well as for strengthening second-year-of-life platforms. As countries continue to adapt immunization schedules to emerging evidence and evolving financing landscapes, such in-person regional learning mechanisms may prove an essential standing mechanism for strengthening policy to best practice in immunization programs and could become more regular and systematic. Figure 7 illustrates the dimensions of the workshop and policy implications of these findings, conceptualizing how regional learning networks influence governance, financing, monitoring and evaluation, and operational preparedness under Gavi 6.0.

Institutionalizing regional collaborative learning platforms in a style similar to the Hexavalent Vaccine Switch Early Adopters Workshop, supporting delivery on Gavi 6.0’s vision of resilient, equitable, and country-owned immunization systems capable of adapting to evolving challenges.

### 4.2. Limitations

This analysis has several limitations. Findings are drawn from a purposive sample of ten early adopter and planning countries participating in the Hexavalent Vaccine Switch Early Adopters Workshop and may not fully capture the diversity of immunization system capacity, governance, and operational contexts across Africa, limiting generalizability. Much of the evidence is based on self-reported country data shared during workshop discussions and planning exercises; while triangulated through peer exchange and facilitation with ideas and contributions written down and contributed anonymously by participants, these data may be subject to social desirability bias, reporting bias, variable quality, limited or incomplete documentation, and preliminary country data results, particularly for early implementation of three months or less and subnational outcomes. In addition, the analysis was conducted within a rapidly evolving global policy and financing context, including the transition to Gavi 6.0. Conclusions would benefit from additional follow-up and more detailed reviews after 6 or 12 months. As guidance, financing modalities, and supply conditions continue to mature, some findings and recommendations may require adaptation. Despite these limitations, the analysis provides timely, practice-oriented insights and a structured framework for regional learning that can be refined as implementation experience expands.

## 5. Conclusions

The Hexavalent Vaccine Switch Early Adopters Workshop illustrates how structured regional collaboration and co-production of knowledge can enhance the quality of vaccine introductions, accelerate decision-making, and strengthen health system resilience. Experiences shared by early adopters demonstrated the operational feasibility and system-level benefits of the hexavalent vaccine switch, while countries planning for introduction gained critical clarity on evidence requirements, financing pathways, logistics, and risk mitigation strategies.

By reframing a technical antigen switch as a platform for shared learning and coordinated planning, the workshop advances a model of practice that extends beyond a single vaccine or event. The co-designed tools and peer-learning mechanisms embedded workshop insights into national systems, supporting more data-driven and adaptive implementation. In doing so, the initiative shows how convenings can evolve from discrete meetings into catalysts for sustained system strengthening.

As Gavi 6.0 places increased emphasis on domestic ownership, multi-year budgeting, and sustainable immunization system performance, such regional learning mechanisms become increasingly important. This model is readily transferable to other antigen switches and vaccine introductions and broader programmatic transitions.

## Figures and Tables

**Table 1:** Forecasting Approaches and Risks Across Participating Countries.

Country	Forecasting Tool	Stock Dynamics	Coexistence Risk	Mitigation Measures
Senegal	Triangulation of census projections, DHIS2 data, and UNICEF/WHO quantification tools; real-time stock monitoring via Logistimo	Tight stock control enabled a clean transition; penta/IPV stocks were closely monitored and depleted before hexa launch	Very low; avoided entirely	Early cancelation of outstanding penta/IPV orders; continuous digital stock monitoring; clean-cut national withdrawal prior to hexa rollout
Mauritania	Census projections and DHIS2 data triangulated with UNICEF/WHO tools	Large inherited penta/IPV stock volumes; parallel use during transition	High; age-phased use of penta/IPV and hexa increased complexity	Age-phased strategy (penta/IPV for older cohorts, hexa for <12 months); enhanced supervision; intensified monitoring during BCU campaigns
Madagascar	Census projections, DHIS2 data, UNICEF/WHO tools	Uneven regional distribution of remaining penta/IPV stocks; staggered depletion	Moderate to high; risk varies by region	Planned staggered regional introduction; emphasis on redistribution, updated tools, and strengthened inventory management
Burundi	Census projections and DHIS2 data triangulated with UNICEF/WHO tools	Limited details pre-introduction; planning phase anticipates tight coordination	Anticipated but avoidable	Strict avoidance of dual administration; alignment with child health platforms; advanced updating of tools and microplans

## Data Availability

Public workshop resources and outputs, including the Hexa Switch Risk Mitigation Catalog and the Health System Adaptation Checklist, are openly available in English and French through the Sabin Vaccine Institute resource page: https://www.sabin.org/resources/insights-from-the-hexavalent-switch-a-process-point-risk-mitigation-compendium/ (accessed on 1 April 2026). Country-specific roadmaps developed during the workshop remain the property of the respective governments and are not publicly available.

## Abbreviations

The following abbreviations are used in this manuscript:

AEFI	Adverse Events Following Immunization
AI	Artificial Intelligence
AMP Afrique	Agence Africaine de Médecine Préventive/African Preventive Medicine Agency
BCU	Big Catch-Up
DHIS2	District Health Information Software 2
DTP	Diphtheria–Tetanus–Pertussis
DTP-HepB-Hib-IPV	Diphtheria–Tetanus–Pertussis–Hepatitis B–*Haemophilus influenzae* type b–Inactivated Poliovirus Vaccine
eBASE	Effective Basic Services Africa
EPI	Expanded Programme on Immunization
Gavi	Gavi, the Vaccine Alliance
GCEI	Groupe Consultatif d’Experts en Immunisation/National Immunization Advisory Structure (Mauritania context)
HeVAC	Hexavalent Vaccine (Switch) Assessment Consortium
HepB	Hepatitis B
Hib	*Haemophilus influenzae* type b
ICC	Inter-agency Coordination Committee
IPV	Inactivated Poliovirus Vaccine
MEL	Monitoring, Evaluation, and Learning
MOH	Ministry of Health
NITAG	National Immunization Technical Advisory Group
OPTIVAC	Optimizing Vaccine Product Choices for Low- and Middle-Income Countries
PIE	Post-Introduction Evaluation
Q1	First Quarter
Q2	Second Quarter
RR2	Second Dose of Measles–Rubella Vaccine
SAGE	Strategic Advisory Group of Experts on Immunization
SOMAPED	Société Mauritanienne de Pédiatrie/Mauritanian Pediatric Society
UNICEF	United Nations Children’s Fund
WHO	World Health Organization
WHO AFRO	World Health Organization Regional Office for Africa
WP	Whole-cell Pertussis
